# The Immune-Antioxidant Trade-Off Mediated by Actinobacteria Drives Niche Differentiation: Physiological and Gut Microbiota Responses of Two Cold-Adapted Brown Frog Species to Contrasting Peak Daily Habitat Temperatures

**DOI:** 10.3390/ani15243604

**Published:** 2025-12-15

**Authors:** Zhenying Lan, Shuang Zhou, Chao Wang, Wanli Liu, Peng Liu

**Affiliations:** College of Life Science and Technology, Harbin Normal University, Harbin 150025, China; 18846513851@163.com (Z.L.); 18845567987@163.com (S.Z.); m18646303344@163.com (C.W.)

**Keywords:** *Rana dybowskii*, *Rana amurensis*, physiological level, gut microbiota, contrasting peak daily habitat temperatures

## Abstract

The fluctuating temperature poses challenges to the survival of amphibians. This study employed two cold-adapted brown frog species, *Rana dybowskii* and *Rana amurensis*, from high-latitude cold regions as research models. We explored the mechanism by which contrasting peak daily habitat temperatures affect their physiological function and symbiotic microbial community. The results indicate that these two cold-adapted brown frog species exhibit a common physiological response of enhanced immune capacity and suppressed antioxidant capacity when subjected to elevated temperatures. However, they demonstrate unique coping strategies and physiological regulatory effects on gut microbiota: *R. dybowskii* activates its immune system by reducing the abundance of Actinobacteria and inhibiting metabolic pathways, but the decrease in Bacteroidetes abundance impairs antioxidant efficacy. On the other hand, *R. amurensis* experiences impairment of antioxidant function due to the regulatory effects of a significant increase in Proteobacteria and Actinobacteria, a marked decrease in Bifidobacterium, and a decline in gut microbiota α-diversity. For the first time, this study reveals the adaptive mechanisms by which two cold-adapted amphibian species respond to the contrasting peak daily habitat temperatures, providing a scientific basis for understanding how ectotherms react to climate change and for predicting their population dynamics.

## Simple Summary

Amphibians, which rely on ambient fluctuating temperature, frequently encounter challenges posed by temperature variability. However, the regulatory mechanisms of contrasting peak daily habitat temperatures on physiology and commensal microbiota remain unclear. By simulating short-term contrasting in the peak daily habitat temperatures before hibernation and after emergence, we systematically analyzed the regulatory effects of temperature changes on the physiological traits and gut microbiota of two cold-adapted brown frog species. The research results indicate that both species of frogs exhibit a common physiological response of enhanced immune capacity and suppressed antioxidant capacity when subjected to elevated temperatures. Furthermore, they show unique coping strategies and physiological regulatory effects on gut microbiota. Despite the convergent response of two species of frogs to the contrasting peak daily habitat temperatures at the physiological level, their differentiated regulation strategies of gut microbiota reflect species-specific adaptive mechanisms and niche partitioning. This finding provides a scientific basis for conserving biodiversity.

## 1. Introduction

Ambient temperature serves as a pivotal factor influencing animal survival, particularly exerting a decisive impact on amphibians that are highly dependent on temperature regulation. Studies have indicated that climate change has led to a significant increase in extinction risk for over 40% of global amphibian species, with their extinction rates ranking among the highest within vertebrates [1,2,3,4]. Spatial and temporal variations in ambient temperature pose different survival challenges for amphibians across various geographical regions and seasons. For instance, large-scale temperature changes (such as latitudinal gradients) significantly affect phenological events: as latitude increases, anuran larvae (e.g., tadpoles) exhibit enhanced tolerance to high temperatures and display more risky behaviors [5,6]. Elevation gain is associated with reduced adult body size, shortened breeding seasons, and an inverted U-shaped relationship with larval growth patterns [7,8]. Additionally, seasonal fluctuations in temperature exert significant regulatory effects on reproductive behaviors, such as the boreal chorus frog (*Pseudacris maculata*), where the frequency of vocal activity significantly increases when the average spring temperature exceeds 5 °C [9]. Although research on small-scale spatial and temporal temperature variations (such as diurnal fluctuations) is relatively scarce, recent evidence suggests that such variations likewise exert important influences on the ecological adaptability of amphibians [10,11,12,13].

In response to fluctuations in ambient temperature, amphibians optimize their survival strategies through physiological adaptations. Among these, immune competence serves as a core indicator of individual viability and is directly regulated by temperature. The immune response intensity of *Pelophylax nigromaculatus* positively correlates with water temperature [14]. An increase in temperature can elevate the proportions of lymphocytes, monocytes, and neutrophils in *Polypedates cruciger* [15]. Conversely, low temperatures impair immune competence by inhibiting immune cell proliferation and complement system activity [16,17]. Furthermore, immune suppression during hibernation in *Lithobates sylvaticus* increases the risk of infection by two parasite species [18]. As the immune systems of amphibians are highly dependent on temperature variations, climate warming may also alter the interaction between the host and the pathogen, thereby affecting the population size [19,20,21,22,23,24,25]. Therefore, temperature, as a crucial environmental factor, significantly affects the dynamic balance among hosts, parasites, and pathogens. This impact may induce cascading changes in amphibian immune system, altering their immune competence. Changes in immune competence may lead to increased sensitivity to pathogens, ultimately exacerbating population survival pressure [15,26]. Fluctuating temperature may also trigger oxidative stress responses. Generally, both excessively high and low temperatures can cause the body to produce and accumulate reactive oxygen species (ROS): in low-temperature environments, particularly during hibernation, antioxidant enzymes such as superoxide dismutase (SOD) and catalase (CAT) exhibit significantly enhanced activity to counteract physiological damage caused by low-temperature exposure [17,27,28,29]. However, if the accumulation of ROS exceeds the scavenging capacity of the antioxidant system, it ultimately causes cellular damage [30,31]. This dynamic balance between oxidation and the antioxidant system is a crucial mechanism for amphibians to maintain physiological homeostasis.

In addition to the physiological responses of the host itself, symbiotic microbial communities play a synergistic role in temperature adaptation [32,33,34]. Ambient temperature drives the spatiotemporal variation in gut microbiota diversity, composition, and function through selective pressure [35,36,37,38,39,40]. For example, high temperatures increase the abundance of *Prevotella* and *Mycobacterium* in the gut of *Lithobates pipiens* tadpoles, while low temperatures promote the proliferation of Proteobacteria [41]. This demonstrates the heterogeneity of temperature effects on the host microbial community [42,43,44]. The microbiota forms a dynamic interaction network with the host, regulating energy metabolism, thermal tolerance, and disease susceptibility through the “microbiota–gut–brain axis” [37,40,44,45,46,47,48]. Metabolites such as short-chain fatty acids (SCFAs) from the microbiota can enhance host antioxidant enzyme activity and reduce inflammatory responses by activating G protein-coupled receptors (GPCRs) and regulating T regulatory (Treg) cell differentiation, thereby enhancing disease resistance [49,50,51,52,53]. For instance, gut microbiota may contribute to the prevention and alleviation of diseases caused by *Batrachochytrium dendrobatidis* or *Batrachochytrium salamandrivorans* [54]. Excessive accumulation of ROS not only directly damages cellular structures but also exacerbates inflammatory responses by altering the composition of the gut microbiota [55]. When ambient temperature changes, an increase in temperature allows the host’s endocrine system to alleviate stress by upregulating antioxidant enzyme activity and promoting the growth of beneficial bacteria, which in turn enhances immune capacity through positive feedback [56]. Conversely, low temperatures may increase the abundance of pathogenic bacteria such as *Citrobacter* and *Pseudomonas*, thereby increasing the risk of immune deficiency [57]. This bidirectional regulatory mechanism suggests a tight functional coupling between the host’s physiological state and the microbial community, which together constitute a “buffering system” in response to environmental stress. This co-evolutionary relationship between the microbiota and host immunity provides a new perspective for understanding survival strategies under environmental stress.

The distinct seasonal climate characteristics in cold regions result in particularly severe fluctuating temperature. Adapting to daily extreme temperature variations has become a core survival challenge for local ectotherms, such as *Rana dybowskii* and *Rana amurensis*. These two cold-adapted brown species of *Rana* are widely sympatric in Heilongjiang Province, yet exhibit divergence in thermal adaptation, habitat selection, trophic niche, and spatial niche, suggesting differential responses to temperature variation [58,59,60]. Although studies have focused on temperature adaptation at the seasonal scale (e.g., metabolic suppression during hibernation) [35,39], the regulatory mechanisms of contrasting peak daily habitat temperatures (e.g., those ranging from 4 °C to 20 °C in April during emergence from hibernation and in October prior to hibernation) on physiology and gut microbiota remain unclear. Such fluctuations may impose dual pressures on individuals already adapted to low temperatures: on the one hand, low-temperature pretreatment may enhance antioxidant capacity; on the other hand, brief high temperatures may disrupt the existing physiological balance, leading to oxidative damage or metabolic disorders. Based on meteorological data from high-latitude sampling sites between 2019 and 2022, this experiment simulated the lower limit (4 °C) and upper limit (20 °C) of the contrasting peak daily habitat temperatures, systematically observing physiological indicators (immune capacity and antioxidant capacity) and changes in gut microbiota structure in two cold-adapted brown frog species. The aims are to elucidate the following scientific questions: (1) How does the physiology of cold-adapted species respond to diurnal contrasting peak daily habitat temperature? (2) Do gut microbiota assist the host in coping with brief high-temperatures stress through functional compensation? (3) Is there niche differentiation in temperature adaptation strategies among different species? The research results will provide theoretical support for the conservation of species in high-latitude cold regions and establish a model foundation for predicting population dynamics under climate change.

## 2. Materials and Methods

### 2.1. Study System, Frogs Collection and Experimental Design

*R. dybowskii* and *R. amurensis* belong to Amphibia, Anura, Ranidae; *Rana*. *R. dybowskii* is mainly distributed in northeast China and the northeast of Inner Mongolia. It often inhabits moist environments such as grassland, pond, farmland and shrub near water sources, and its typical habitat is broad-leaved forest or mixed broad-leaved forest. *R. amurensis* is the most widely distributed amphibious species in the Palaearctic. Its distribution and habitat are very similar to those of *R. dybowskii*, and it often lives below 600 m above sea level in low-mountain coastal areas with humid climate, dense vegetation and overgrown weeds [61].

The experiments were conducted in strict accordance with the Institutional Animal Care and Use Committee (IACUC) ethical guidelines of Harbin Normal University (HNUARIA2022004). No endangered or protected species were involved in this study, and all efforts were made to minimize animal suffering. In October 2023, eighteen individuals per species of *R. dybowskii* and *R. amurensis* were collected from Acheng City and Hailin City. The two species were weighed (*R. dybowskii*: female 32.01 ± 1.23 g, male 18.23 ± 0.75 g; *R. amurensis*: female 23.32 ± 0.98 g, male 12.34 ± 2.37 g). Both species were housed individually (one per container) in identical plastic containers (28 cm × 19 cm × 14 cm) to preclude inter-individual interference. The housing environment comprised aerated groundwater at a depth of 5 cm, a 15° slope constructed from sterilized stones, pH 6.8 ± 0.45, dissolved oxygen concentration ≥ 5 mg/L, a 12:12 h light–dark cycle, and daily replacement of 20% of the water volume (Figure S1A) [58].

Both species experience pronounced daily maximum temperature amplitude following April emergence and prior to October hibernation, with observational data from 2019–2022 from the National Meteorological Science Data Center (http://data.cma.cn/site/index.html, accessed on 11 December 2025), indicating that their daily maximum temperature extremes range between 4 °C and 20 °C (Figure S1B). Based on documented biological characteristics of reduced feeding or fasting in frogs following April emergence and prior to October hibernation, a three-week fasting period was implemented in this study [62]. Based on contrasting peak daily habitat temperatures, *R. dybowskii* and *R. amurensis* individuals of similar body mass and in good health condition were each divided into two temperature treatment groups (4 °C and 20 °C), with 9 individuals per group (4 females and 5 males). To avoid the influence of other variables, *R. dybowskii* and *R. amurensis* in the two temperature groups were consistent in all environments except temperature. 

### 2.2. Immune Capacity and Antioxidant Capacity Assays

#### 2.2.1. Determination of Phytohemagglutinin (PHA) Response Intensity

The *R. dybowskii* and *R. amurensis* were respectively weighed. Literature reports indicate that the PHA response peaks at 12 h, which was further confirmed by our pilot experiments (Figure S1C,D) [63]. The thicknesses of the right and left posterior sole-pads were measured with a digital thickness gauge (Micrometer Mitutoyo, Kawasaki, Japan, 0–25 mm) before injection of either PHA or phosphate-buffered saline (PBS) following the methodology of previous studies [14]. Immediately after these measures, 5 μL of PHA solution (2.5 mg/mL) was injected in the posterior left hind leg (treatment) and the same volume of PBS was injected into the right hind leg (control). The thickness of the right and left hind legs was measured 12 h after injections. The swelling in response to PHA (treatment) or PBS (control) was estimated from the proportional increase in thickness in the posterior hind legs before and after the injection. The PHA test does not cause any negative health effects and the reaction stimulated by the PHA disappears within 48 h after the injection, as has been previously shown in other amphibian species [63,64].

#### 2.2.2. Collection of Experimental Samples

Based on the literature-reported 3-day metabolic stabilization period, the frogs were weighed after completing a 3-day acclimation period [65]. Gauze was laid out neatly in a glass dryer, and subsequently, a cotton ball, saturated with a blend of ether and alcohol, was positioned underneath to induce anesthesia in the frog. The frogs were then put to death via double pithing and decapitation in accordance with ethical regulations [66]. Anatomical experiments were then conducted to rapidly excise the liver, stomach, intestines, and heart. After the tissue surface was blotted with absorbent paper to remove excess liquid, the organs were weighed and transferred to centrifuge tubes. The centrifuge tubes containing the liver, stomach, and intestine were promptly frozen in liquid nitrogen at −80 °C for subsequent analysis.

#### 2.2.3. Preparation of Blood Smears and Enumeration of Lymphocytes

Following pithing euthanasia, the frogs were placed supine on dissecting boards. Blood (3 μL) was collected using a pipette and dropped onto one end of a microscope slide, then rapidly smeared to create uniform blood films. The dried smears were stained with Rapid Wright-Giemsa stain solution (D010) from Nanjing Jiancheng Bioengineering Institute (Nanjing, Jiangsu Province, China). After staining and drying, white blood cells were examined under a SMART series biological microscope (40× objective, 10× eyepiece; Chongqing, Sichuan Province, China), selecting smears with good staining quality and higher leukocyte counts, then mounted with resin. The mounted smears were observed under the microscope, and a manual differential cell counter was used to determine the percentages of lymphocytes (Lym) per 200 white blood cells. Stained cells were photographed using a MOTICAM Pro S5 Lite microscopic (Xiamen, Fujian Province, China) imaging system. Each temperature group had 5 replicate blood smears.

#### 2.2.4. Determination of Enzyme Activity

Enzyme activity assay kits from Nanjing Jiancheng Bioengineering Institute (Nanjing, Jiangsu Province, China) were used according to the manufacturer’s instructions. Liver samples (about 0.05 g) were homogenized in 0.65% normal saline and centrifuged at 5000 g at 4 °C for 15 min. The supernatants were collected immediately for the subsequent analyses. Lysozyme (LSZ, A050-1-1) was measured by turbidimetry, total protein (TP, A045-2-1) by the Coomassie brilliant blue method, superoxide dismutase (SOD, A001-3-2) by the WST-1 method, catalase (CAT, A007-1-1) by the ammonium molybdate method, and malondialdehyde (MDA, A003-1-1) by the TBA method. The content of each parameter was then calculated according to the formulas provided in the kit instructions to assess the immune function and antioxidant capacity of the two brown frog species.

### 2.3. Guts Collection and Gut Microbiota Analysis

#### 2.3.1. Library Sequencing and Quality Filtering

After the physiological metrics were tested, the entire intestinal tract of *R. dybowskii* and *R. amurensis* was subjected to gut microbiota analysis. DNA extraction, amplification, and sequencing were conducted by PersonalBio Biotechnology Co., Ltd. (Shanghai, China). Total genomic DNA samples were extracted using the MagBeads FastDNA Kit for Soil (116564384) (MP Biomedicals, Santa Ana, CA, USA). PCR amplification of the V3–V4 region of the bacterial 16S rRNA gene was performed using the forward primer 338F (5′-ACTCCTACGGGAGGCAGCA-3′) and the reverse primer 806R (5′-GGACTACHVGGGTWTCTAAT-3′). The products were purified, quantified, and normalized to generate the sequencing library. The Illumina NovaSeq 6000 platform (San Diego, CA, USA) was used to sequence the qualified library. Base calling was used to convert the original image data files obtained via high-throughput sequencing (Illumina NovaSeq and other sequencing platforms) into the original sequence reads. The sequenced raw reads were filtered using Trimmomatic v0.33 software [67]. Then, cutadapt 1.9.1 software was used to identify and remove the primer sequences to obtain clean reads without the primer sequences [68].

#### 2.3.2. DADA2 Denoising and Taxonomy Classification

Denoising was performed using QIIME2 2020.6, following the pipeline proposed by Maki et al. [69]. All of the parameter settings are in the drop-down menu for each analytical step. The first entity is the default setting. Sequences were then quality filtered, denoised, merged and chimera removed using the DADA2 0.99.8 plugin [70]. Finally, a total of 1,319,155 and 1,954,879 valid sequences of the hypervariable V3–V4 region of the 16S rRNA gene were obtained from the fecal samples for *R. dybowskii* and *R. amurensis*, respectively. By using the Greengenes database, the ASV feature sequences were compared with the reference sequences in the database to obtain the taxonomic information corresponding to each ASV [71]. ASVs with abundance values < 0.001% of the total sequencing volume of the entire sample were removed, and the abundance matrix with rare ASVs removed for subsequent analysis was used. Principal component analysis (PCA) was performed using the Bray–Curtis distance metric (R v4.4.2). Based on the ASV classification and taxonomic status identification results, the specific species composition of each sample was obtained at all taxonomic levels from phylum to species.

#### 2.3.3. Diversity Index, Taxonomy Differential Abundance Analysis and Microbiome Function Prediction

Alpha diversity indices (Chao1, Faith pd, Goods coverage, Shannon index, Simpson index, Pielou e, and Observed species) and beta diversity metrics (Bray–Curtis dissimilarity and weighted UniFrac distance) were calculated using QIIME2, with the ASV (Amplicon Sequence Variant) table rarefied to 32,335 reads per sample. Nonmetric multidimensional scaling (NMDS) based on the Bray–Curtis distance was used to determine the variations in community diversity among different samples. Using the Mann–Whitney U test, we compared the changes in the relative abundance of the gut microbiota composition in *R. dybowskii* and *R. amurensis*.

The unique and shared ASVs between the groups were plotted using a Venn diagram. EasyMAP (http://easymap.cgm.ntu.edu.tw/, accessed on 11 December 2025) was applied linear discriminant analysis (LDA) effect size (LEfSe) to identify microbes with significant differences between groups from KEGG level 1~level 2 [72]. The default alpha value for a Wilcoxon test between different sub-groups was 0.05. The default threshold on the absolute value of logarithmic LDA score was 2 [73]. The default alpha value for Kruskal–Wallis test among statistical groups was 0.05. For predicting microbe function, Greengenes database (version 13_8, 99% OTU) and VSEARCH (v2.13.4) were applied for close-reference clustering, which ensures that the representative sequences map to the Greengenes database [74]. PICRUSt (Phylogenetic Investigation of Communities by Reconstruction of Unobserved States) was used for the mapped Greengenes IDs to identify the corresponding functions in the KEGG (Kyoto Encyclopedia of Genes and Genomes) database [75].

### 2.4. Statistical Analysis

Statistical analyses were conducted using SPSS software (version 27.0; SPSS, Inc., Chicago, IL, USA). Normality and homogeneity were evaluated using the Kolmogorov–Smirnov and Levene’s tests, respectively. The physiological indices of *R. dybowskii* and *R. amurensis* were analyzed by single factor analysis of variance (ANOVA) and LSD, and the images were drawn and processed by GraphPad Prism 10.4. When 0.01 < *p* < 0.05 is considered to have a significant difference, *p* < 0.01 is considered to have an extremely significant difference, and *p* > 0.05 is considered to have no significant difference. In this study, sex differences were not significant; therefore, sex was excluded as a factor, and only temperature effects were examined (Table S1). All data are expressed as the mean ± standard error. Through the Mantel test, key microbiota taxa involved in differential pathways of gut microbiota were categorized into two groups based on immune capacity and antioxidant capacity. Specifically, Mantel’s r and Mantel’s *p* values were utilized to assess the correlations.

## 3. Results

### 3.1. Immune Capacity and Antioxidant Capacity

**Figure 1:**
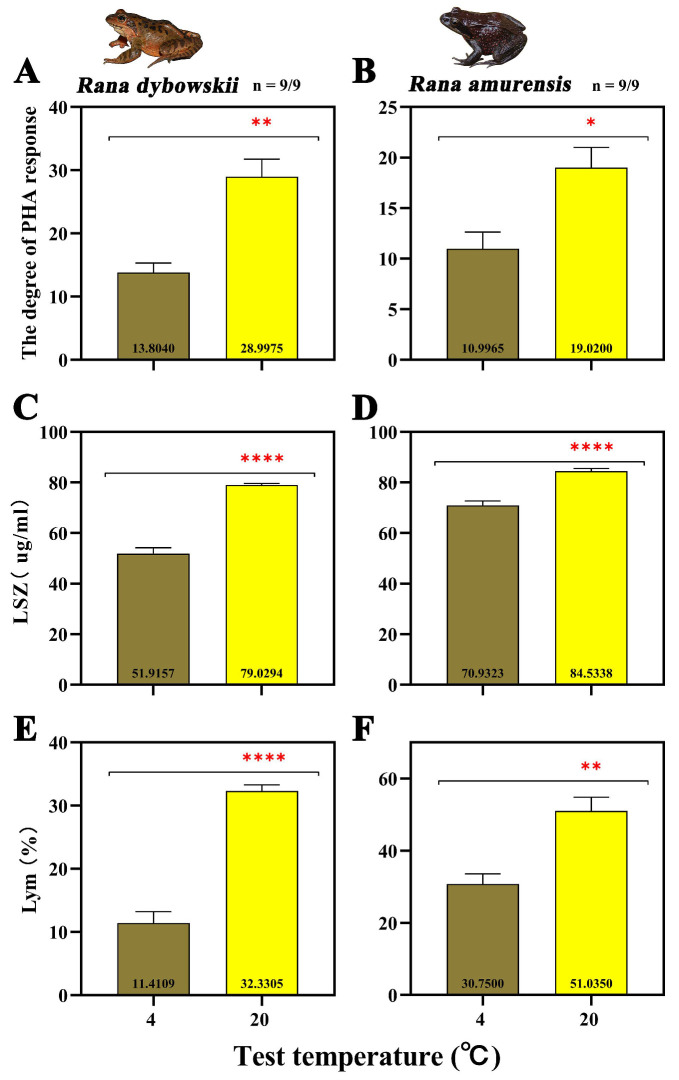
The degree of PHA response (**A**,**B**), LSZ (**C**,**D**), and Lym (**E**,**F**) of *R. dybowskii* and *R. amurensis* at different temperatures. Significant group differences are denoted by asterisks. * stands for 0.01 < *p* ≤ 0.05, ** stands for 0.001 < *p* ≤ 0.01, and **** stands for *p* < 0.000.

The degree of PHA response of both frog species increased significantly with increasing temperature (*R. dybowskii*: *F* = 23.422, *p* = 0.008; *R. amurensis*: *F* = 9.655, *p* = 0.036) (Figure 1A,B). The LSZ content of both frog species increased significantly at 20 °C (*R. dybowskii*: *F* = 132.309, *p* = 0.000; *R. amurensis*: *F* = 43.276, *p* = 0.000) (Figure 1C,D). For *R. dybowskii*, the percentage of Lym in white blood cells increased significantly with the increase in temperature (*F* = 104.074, *p* = 0.000) (Figure 1E). For *R. amurensis*, the percentage of Lym in white blood cells decreased significantly with the increase in temperature (*F* = 18.471, *p* = 0.003) (Figure 1F).

**Figure 2:**
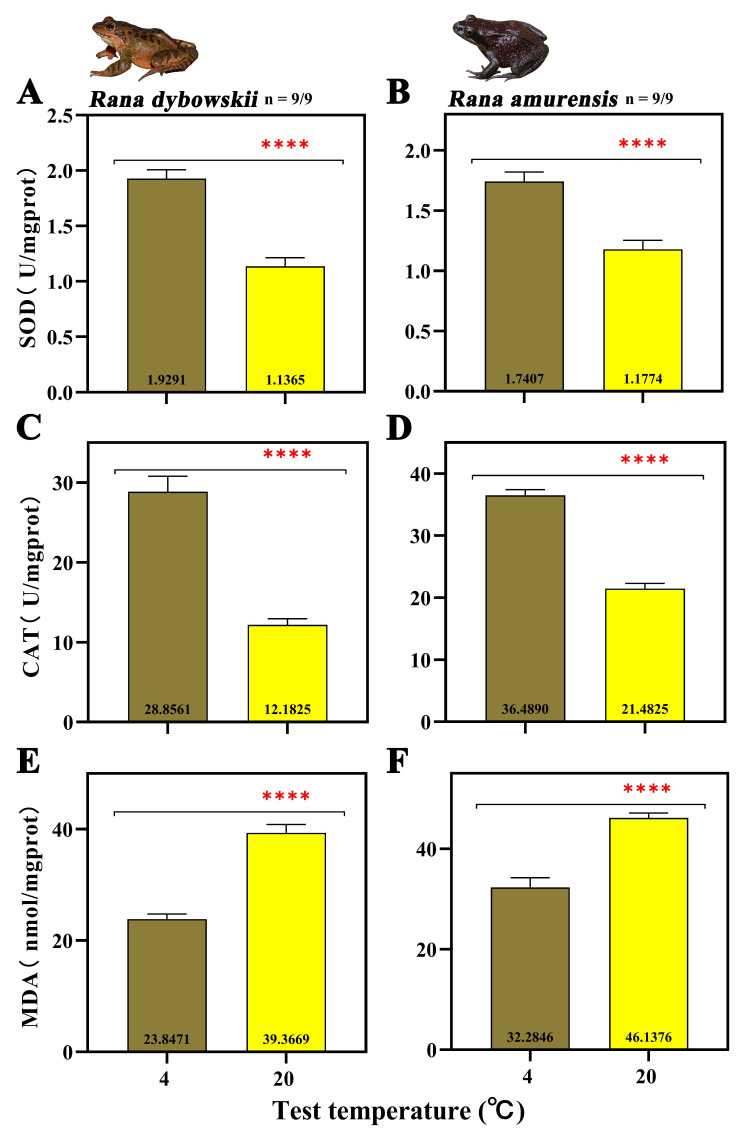
The activity of SOD (**A**,**B**), CAT (**C**,**D**), and MDA (**E**,**F**) of *R. dybowskii* and *R. amurensis*. Significant group differences are denoted by asterisks. **** stands for *p* < 0.000.

At 20 °C, the SOD activity decreased significantly (*R. dybowskii*: *F* = 52.699, *p* = 0.000; *R. amurensis*: *F* = 26.647, *p* = 0.000) (Figure 2A,B). For both species, CAT activity decreased significantly (*R. dybowskii*: *F* = 64.389, *p* = 0.000; *R. amurensis*: *F* = 152.168, *p* = 0.000) (Figure 2C,D) and MDA activity increased significantly with increasing temperature (*R. dybowskii*: *F* = 80.335, *p* = 0.000; *R. amurensis*: *F* = 41.383, *p* = 0.000) (Figure 2E,F).

### 3.2. Gut Microbiota Analysis

#### 3.2.1. Alpha and Beta Polymorphic Index Analysis

**Figure 3:**
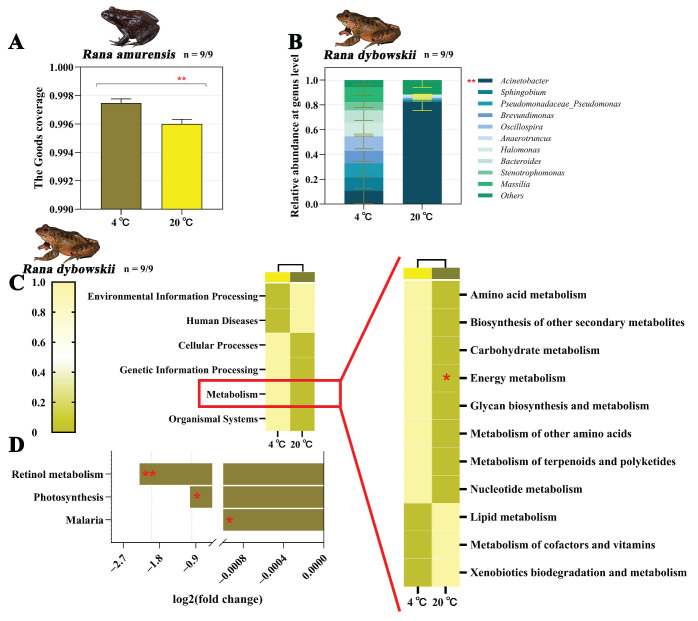
The alpha diversity index of *R. amurensis* (**A**), bacteria composition of intestinal flora of *R. dybowskii* (**B**), functional classifications and functionally predicted KEGG pathways of *R. dybowskii* (**C**,**D**) at different temperatures. Significant group differences are denoted by asterisks. * stands for 0.01 < *p* ≤ 0.05, and ** stands for 0.001 < *p* ≤ 0.01.

We obtained 1776, 1526, 4717, and 4497 ASVs from 4 °C and 20 °C of *R. dybowskii* as well as 4 °C and 20 °C of *R. amurensis*, respectively. The alpha polymorphic index (goods coverage index) of *R. amurensis* showed significant difference between different temperature treatments (*Z* = −2.782, *df* = 1, *p* = 0.005) (Figure 3A). There was no significant difference in the alpha polymorphic index between *R. dybowskii* and *R. amurensis* at different temperatures (all *p* > 0.05) (Figure S2A,B). Also, there was no significance in the beta polymorphic index difference between the groups of *R. dybowskii* (*R*^2^ = 0.075, *p* = 0.163) (Figure S3A) and *R. amurensis* (*R*^2^ = 0.123, *p* = 0.055) (Figure S3C).

#### 3.2.2. Composition Analysis of Gut Microbiota

For *R. dybowskii*, the ASVs obtained from the samples consisted of 6 phyla, 14 classes, 19 orders, 104 families, 191 genera, and 75 species. The three florae with the highest phylum level abundance were Proteobacteria, Firmicutes and Bacteroidetes (Figure S4A). Abundance of the highest in the class three flora were Gammaproteobacteria, Clostridia and Alphaproteobacteria (Figure S4C). The three florae with the highest abundance at the order level were Pseudomonadales, Clostridiales and Enterobacteriales (Figure S4E). The three florae with the highest level of family abundance were Moraxellaceae, Enterobacteriaceae and Ruminococcaceae (Figure S4G). The three florae with the highest level of genus abundance were *Acinetobacter*, *Sphingobium* and *Pseudomonadaceae Pseudomonas*. It should be noted that *Acinetobacter* content was significantly different at different temperatures (*Z* = −3.230, *df* = 1, *p* = 0.001) (Figure 3B and Figure S4I). The three florae with the highest level of species abundance were *Acinetobacter johnsonii*, *Acinetobacter schindleri*, and *Sphingobium yanoikuyae* (Figure S4K).

For *R. amurensis*, the ASVs obtained from the samples consisted of 4 phyla, 12 classes, 167 orders, 269 families, 369 genera and 132 species. The three florae with the highest phylum level abundance were Firmicutes, Proteobacteria and Bacteroidetes (Figure S4B). Abundance of the highest in the class three flora were Bacilli, Gammaproteobacteria and Bacteroidia (Figure S4D). The three florae with the highest abundance at the order level were Lactobacillales, Bacteroidales and Clostridiales (Figure S4F). The three florae with the highest level of family abundance were Leuconostocaceae, Enterobacteriaceae and Pseudomonadaceae (Figure S4H). The three florae with the highest level of genus abundance were *Weissella*, *Pseudomonadaceae Pseudomonas* and *Bacteroides* (Figure S4J). The three florae with the highest level of species abundance were *Weissella paramesenteroides*, *Pseudomonas veronii* and *Silene vulgaris* (Figure S4L).

#### 3.2.3. Differential Analysis and Functional Annotation of Microbial Community

**Figure 4:**
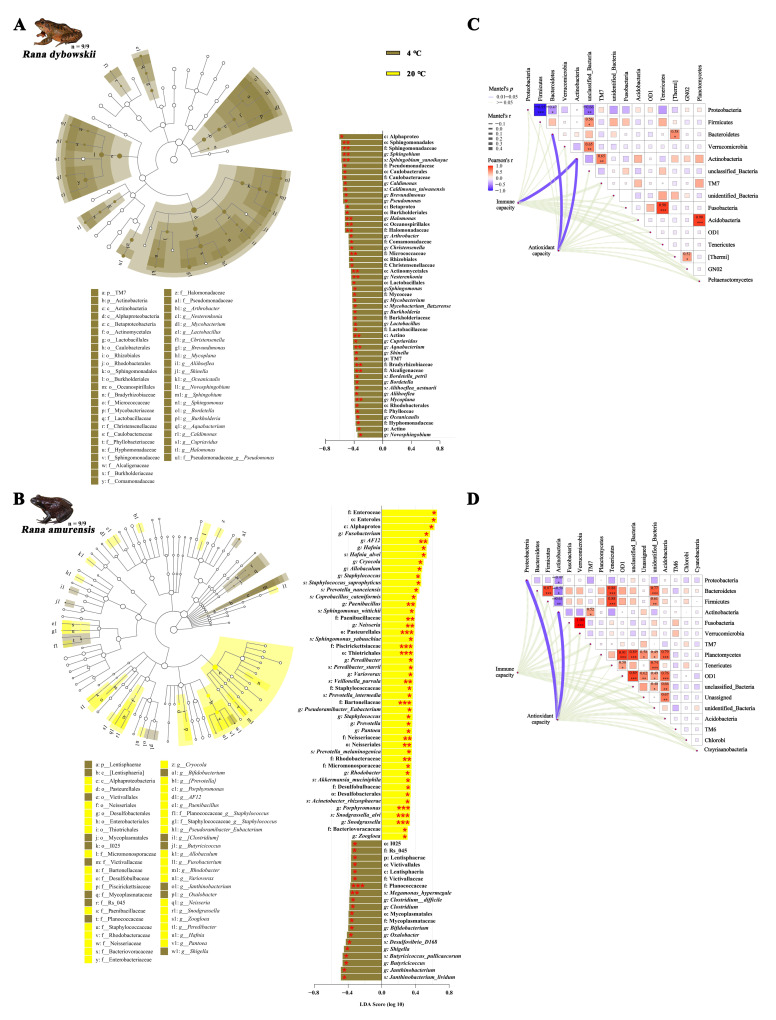
The differences in taxa determined by linear discriminative analysis of effect size (LEfSe) and the correlation analysis of *R. dybowskii* (**A**,**C**) and *R. amurensis* (**B**,**D**). * stands for 0.01 < *p* ≤ 0.05, ** stands for 0.001 < *p* ≤ 0.01, and *** stands for *p* < 0.001.

The 4 °C and 20 °C shared 668 ASVs, but 1108 and 858 ASVs were unique to 4 °C and 20 °C of *R. dybowskii*, respectively. In addition, 4 °C and 20 °C shared 1443 ASVs (Figure S5A), but 3274 and 3054 ASVs were unique to 4 °C and 20 °C of *R. amurensis*, respectively (Figure S5B). LEfSe was used to detect variations in the relative abundance of microbiota at different hierarchies to further identify shifts in the composition of gut microbes in different frogs. The results showed that 52 gut microbiota taxa (2 phyla, 3 classes, 8 orders, 14 families, 20 genera, and 5 species) of *R. dybowskii* were more abundant at 4 °C. The single class (Actino), 3 orders (Actinomycetales, Oceanospirillales, and Sphingomonadales), 5 families (Alcaligenaceae, Bradyrhizobiaceae, Micrococcaceae, Halomonadaceae, and Sphingomonadaceae), 5 genera (*Mycoplana*, *Aquabacterium*, *Nesterenkonia*, *Halomonas*, and *Sphingobium*), and 1 species (*Sphingobium yanoikuyae*) were the major taxons contributing to these differences (all LDA scores > 2, *p* < 0.01) (Figure 4A). Sixty-seven gut microbiota taxa differed in abundance, among which 47 taxa (1 classes, 5 orders, 10 families, 18 genera, and 13 species) were more abundant at 20 °C and 20 taxa (1 phylum, 1 class, 3 orders, 4 families, 7 genera, and 4 species) were more abundant at 4 °C of *R. amurensis*. The 3 orders (Neisseriales, Thiotrichales, and Pasteurellales), 5 families (Planococcaceae, Neisseriaceae, Bartonellaceae, Piscirickettsiaceae, and Paenibacillaceae), 6 genera (*Snodgrassella*, *Porphyromonas*, *Rhodobacter*, *Neisseria*, *Paenibacillus*, and *AF12*), and 3 species (*Megamonas hypermegale*, *Snodgrassella alvi*, and *Veillonella parvula*) were the major taxa contributing to these differences at 20 °C of *R. amurensis* (all LDA scores > 2, *p* < 0.001) (Figure 4B). 

In the functional prediction, only *R. dybowskii* showed significant changes. The pathways associated with the Energy metabolism were significantly upregulated at 4 °C of *R. dybowskii* (*F*_1,16_ = 4.983, *p* = 0.040) (Figure 3C). Also, under the condition of 4 °C, the ko05144-Malaria (*p* = 0.022), ko00195-Photosynthesis (*p* = 0.038) and ko00830-Retinol metabolism (*p* = 0.010) pathways were significantly upregulated for *R. dybowskii* (Figure 3D).

### 3.3. Correlation Analysis and Redundancy Analysis

For *R. dybowskii*, the antioxidant capacity was positively correlated with Bacteroidetes (r = 0.237, *p* = 0.040) and Actinobacteria (r = 0.218, *p* = 0.032), and the immune capacity was positively correlated with Actinobacteria (r = 0.294, *p* = 0.012). Proteobacteria was positively correlated with Firmicutes, Bacteroidetes and unclassified Bacteria. Firmicutes was negatively correlated with unclassified Bacteria. Bacteroidetes was negatively correlated with Thermi. Verrucomicrobia was negatively correlated with unclassified Bacteria. Actinobacteria was negatively correlated with TM7. Fusobacteria were negatively correlated with Tenericutes. Thermi was negatively correlated with GN02 (all *p* < 0.05) (Figure 4C).

However, for *R. amurensis*, the antioxidant capacity was positively correlated with Proteobacteria (r = 0.217, *p* = 0.030) and Actinobacteria (r = 0.177, *p* = 0.038). Proteobacteria was positively correlated with Actinobacteria. Bacteroidetes was negatively correlated with Firmicutes, Tenericutes and unidentified Bacteria, but positively correlated with Actinobacteria. Firmicutes was negatively correlated with Tenericutes and unidentified Bacteria, but positively correlated with Actinobacteria. Actinobacteria was negatively correlated with TM7. Fusobacteria were negatively correlated with Verrucomicrobia. Planctomycetes was negatively correlated with OD1, unclassified Bacteria, Unassigned, unidentified Bacteria and Acidobacteria. Tenericutes was negatively correlated with OD1 and unidentified Bacteria. OD1 was negatively correlated with unclassified Bacteria, Unassigned, unidentified Bacteria and Acidobacteria. Unclassified Bacteria was negatively correlated with unidentified Bacteria and Acidobacteria. Unassigned was negatively correlated with Acidobacteria (all *p* < 0.05) (Figure 4D).

## 4. Discussion

Research on the adaptive mechanisms of ectotherms to extreme temperatures has primarily focused on maintaining physiological homeostasis during hibernation. However, there remains a cognitive gap regarding their response mechanisms to contrasting temperatures during the transition between hibernation and active periods. In this study, we focused on two cold-adapted brown frog species, *R. dybowskii* and *R. amurensis*, and systematically uncovered for the first time the regulatory effects of contrasting peak daily habitat temperatures before hibernation and after emergence from hibernation on their physiological levels and gut microbiota structure. Notably, Actinobacteria was found to play a pivotal role in the interaction between these two factors. Furthermore, variations in the gut microbiota of cold-adapted frog species within the same genus at the same latitude exhibited differences, indicating niche differentiation.

### 4.1. Dual Effects of Contrasting Peak Daily Habitat Temperatures on Physiological Homeostasis

Ambient temperature, as a critical ecological factor, directly influences the immune and antioxidant systems of ectotherms. This study found that two cold-adapted brown frog species exhibited significant physiological adaptation differentiation in response to contrasting peak daily habitat temperatures. When the ambient temperature rose, their innate immune capacity demonstrated a positive response (Figure 1A,B), while the antioxidant system suffered significant inhibition (Figure 2A,B). This seemingly contradictory physiological response pattern actually reflects an optimized choice of energy allocation strategies.

Regarding immune regulation, an increase in peak daily habitat temperatures triggers the non-specific innate immune system, resulting in synchronous enhancements of inflammatory responses, humoral factors, and cellular components [76,77]. This rapid response mechanism facilitates pathogen clearance, which is directly related to the increased environmental pathogenic load caused by warming [15,78,79]. Although this differs from many studies showing that warming reduces the immunity of ectotherms, it may be due to the fact that the tolerance temperature threshold has not been exceeded [21]. However, this immune enhancement may incur adaptive costs. Upon emerging from hibernation, the two cold-adapted brown frog species immediately entered the breeding season. At this time, the heightened immune capacity of ectotherms can decrease reproductive fitness, particularly in males, thereby adversely affecting population sustainability [80]. Additionally, in low-temperature environments, the immune capacity of the two cold-adapted brown frog species was weakened, which aligns with the inhibitory effect on pathogens at lower ambient temperatures, thereby reducing the burden on the immune system [16,17]. This provides a new perspective for explaining seasonal fluctuations in amphibian population dynamics.

In the context of oxidative stress, fluctuating diurnal high temperatures exert adverse effects on organisms. Studies have found that higher peak daily habitat temperatures lead to a decline in the antioxidant defense mechanisms of two cold-adapted brown frog species, impairing their ability to effectively scavenge excess ROS, thereby resulting in the accumulation of oxidative damage (Figure 2E,F). During cellular metabolism, the production and scavenging of ROS are usually in a dynamic balance. Moderate levels of ROS play a crucial role in maintaining intracellular environmental stability, signal transduction, and cellular function. However, external environmental pressures may disturb this balance, leading to elevated ROS levels and subsequent damage to biological molecules such as DNA, lipids, and proteins, resulting in cytotoxicity [31]. In this study, elevated temperatures increased metabolic rates and oxygen consumption, leading to the production of excessive ROS. Meanwhile, the activities of CAT and SOD in two cold-adapted brown frog species decreased with increasing temperatures. This may be due to irreversible damage caused by rapid temperature increases, which disrupts the integrity of the antioxidant system and inhibits its ability to scavenge excess ROS in the liver. This finding is consistent with previous research results on other anurans under high temperature conditions [29,30,31,81]. The termination of hibernation also affected antioxidant capacity. According to the “Preparation for Oxidative Stress” (POS) theory, animals consume glycogen during hibernation to enhance their antioxidant defense in preparation for subsequent oxidative stress [17,82,83,84]. However, this significant consumption during hibernation leads to an inability to maintain corresponding antioxidant levels after spring recovery, making it impossible to promptly scavenge ROS caused by contrasting peak daily habitat temperatures [85]. Furthermore, the accumulation of oxidative damage before hibernation may bring additional burdens to subsequent hibernation, ultimately reducing the survival rates of the two cold-adapted brown frog species [86,87]. In summary, the high temperature environment resulting from contrasting peak daily habitat temperatures activates the immune system and causes oxidative damage in the two cold-adapted brown frog species, enabling them to adapt to changes in their habitat.

Despite the extremely similar habitats and sympatric distribution of the two cold-adapted brown frog species, they exhibit a certain degree of niche separation. Consistent with previous research results, under the same ambient temperature, the immune and antioxidant levels of *R. amurensis* are higher than those of *R. dybowskii* [59]. However, under corresponding conditions of contrasting peak daily habitat temperatures, *R. dybowskii* may exhibit more intense physiological responses. *R. dybowskii*, as a cold-adapted amphibian, has lower cold tolerance than *R. amurensis* and may be more sensitive to frequent and rapidly fluctuating ambient temperatures [60].

### 4.2. Dynamic Response of Gut Microbiota to Contrasting Peak Daily Habitat Temperatures

As the “second genome” of the host, the gut microbiota exhibits unique niche differentiation characteristics in two cold-adapted brown frog species when facing contrasting peak daily habitat temperatures [55,88,89]. At the level of microbial diversity, the α-diversity of *R. amurensis* decreased with increasing temperature, while that of *R. dybowskii* remained stable (Figure 3A and Figure S2). This difference may originate from species-specific differentiation in cold tolerance: as a cold-adapted species more suited to severe cold, *R. amurensis* may exhibit a less adaptive response to high temperatures [60,79].

This study reveals that the core microbial communities (Proteobacteria, Firmicutes, and Bacteroidetes) of the two cold-adapted brown frog species maintain high structural stability, constituting their core microbial communities [90]. These microbial communities, which are highly similar in composition and function, are considered to be intrinsic to amphibians [91]. As the primary digestive phyla, Firmicutes and Bacteroidetes work together in the digestion of food proteins, thereby promoting the absorption of nutrients [92,93,94]. The study found that the ratio of Bacteroidetes/Firmicutes in the gut microbiota decreased with increasing temperature (*R. dybowskii*: 4 vs. 20 = 0.18 vs. 0.15; *R. amurensis*: 4 vs. 20 = 0.43 vs. 0.19). Given the positive correlation between Bacteroidetes/Firmicutes and the host’s body weight or body mass index (BMI), this suggests changes in the host’s energy metabolism pattern, with increased energy consumption [95,96]. The simulation time points selected for this study were during the hibernation preparation period and the emergence period (April and October). During these times, metabolic activity slows down, and the animals are in a fasting or reduced feeding state, unable to obtain sufficient energy from the external environment. This is consistent with the influence of diet on the composition of the gut microbiota [97,98]. However, if exposed to higher temperatures for a longer period, it may lead to weight loss and adversely affect survival [87,99]. Additionally, this study detected the presence of Verrucomicrobia and Tenericutes in two cold-adapted brown frog species. Although their relative abundances are low and they do not show significant changes with increasing temperature, their presence also suggests metabolic changes [92]. Meanwhile, Cyanobacteria were found in the guts of two cold-adapted brown frog species, although this type of microorganism is usually found in the guts of tadpoles rather than in adult food [38]. This may be due to the brown frogs inadvertently ingesting them while moving and foraging in shrubbery near the shore during migration from high-altitude areas to water sources before hibernation [62,91]. Furthermore, *R. amurensis* also exhibited the presence of Fusobacteria (Figure S4B). In insectivorous animals, Fusobacteria work synergistically with Bacteroidetes, Firmicutes, and Proteobacteria to help the host digest chitin from insects [100]. These taxa related to nutrition and metabolism exhibit certain correlations (Figure 4C,D) and they may have complex synergistic effects when functioning [48,101]. Finally, although the three dominant phyla at the phylum level were consistent with the seasonal changes in the gut microbiota of the two cold-adapted brown frog species reported in previous studies, there were differences in their proportions, reflecting the influence of seasonal changes on the gut microbiota [38,39]. However, at the genus level, *R. dybowskii* exhibited an increase in the abundance of *Acinetobacter* with increasing temperature (Figure 3B), which, along with the increase in opportunistic pathogens in the Proteobacteria phylum, may be important inducer of continuous activation of the host immune system [91,102].

The LEfSe analysis further revealed the downregulation of 14 beneficial bacterial groups and 24 pathogenic bacterial groups in *R. dybowskii* in response to increased temperatures (Figure 4A). Under the same conditions, in *R. amurensis*, the downregulation of 10 beneficial bacterial groups and 6 pathogenic bacterial groups, as well as the significant enrichment of 24 beneficial bacterial groups and 14 pathogenic bacterial groups (Figure 4B), became apparent. With the elevation of ambient temperature, the pathogenic bacteria mainly showed a significant decrease in one phylum (Actinobacteria) and two classes (Alphaproteobacteria and Betaproteobacteria) in *R. dybowskii*. This reduction contributed to some extent to the maintenance of the host’s healthy state and enhanced its adaptability [103,104]. Conversely, *R. amurensis* primarily showed a reduction in one probiotic phylum (Lentisphaerae) and an increase in one pathogenic class (Alphaproteobacteria). This, coupled with decreased diversity, could lead to gut ecological imbalance and potentially adversely affect the host’s immune capacity [104,105].

Finally, with the increase in ambient temperature, the functional group abundance of *R. dybowskii* experienced significant adjustments (Figure 3C). As temperature increases, metabolic functions undergo downregulation in response to the elevation in body temperature [106]. This is associated with greater fluctuations in body temperature, a lower thermoregulatory capacity compared to *R. amurensis*, and limited food resources available to *R. dybowskii* [35,107,108]. This alteration in metabolic functions further led to the downregulation of immune-related pathways such as ko05144-Malaria and ko00830-Retinol metabolism (Figure 3D), inhibiting parasite metabolism and activating the host’s defense mechanisms, thereby enhancing the immune capacity of the organism [109,110]. However, if energy intake remained relatively constant, allocating more to the immune system might reduce investment in subsequent reproductive/hibernation events [111]. Notably, adult individuals of both species that experienced short-term contrasting peak daily habitat temperatures before hibernation and after emergence exhibited gut microbiota characteristics similar to those during the tadpole stage [38,112]. This change may be related to their higher vulnerability to thermal environments during this period [113]. In summary, the gut microbiota of brown frog species before hibernation and after emergence were still adapting to cold habitats, and contrasting peak daily habitat temperatures had numerous adverse effects on their gut microbiota balance [114]. This “structurally conserved-functionally plastic” response pattern provides a compelling explanation for the microbiological evidence of host phenotypic plasticity.

### 4.3. Regulatory Mechanisms of the Gut Microbiota–Host Interaction Network

The co-evolution of gut microbiota communities and host physiological systems has established complex feedback regulatory networks during temperature adaptation [37,79,115]. This study revealed that Actinobacteria play a pivotal regulatory role in this network. The enhancement of immune capacity in *R. dybowskii* was associated with a decrease in Actinobacteria abundance; whereas a decline in antioxidant capacity was correlated with a reduction in the abundance of Bacteroidetes and Actinobacteria (Figure 4C). In *R. amurensis*, the decrease in antioxidant capacity is related to changes in the abundance of Proteobacteria and Actinobacteria (Figure 4D). The Actinobacteria and their subordinate pathogenic populations in the gut of *R. dybowskii* significantly decreased with increasing temperature, which may reflect an enhancement of host immunity (Figure 4A) [53,116,117]. However, the reduction in probiotic Bacteroidetes at higher temperatures may decrease food utilization efficiency, posing additional pressure on *R. dybowskii* [49], which already faces a scarcity of food resources before emerging from hibernation and entering hibernation [118]. This decrease in energy supplementation may correspond to an inability to clear more oxidative damage. Meanwhile, it is known that SCFAs enhance antioxidant enzyme activity by activating GPCR signaling pathways. Thus, a decrease in their concentration may exacerbate oxidative damage effects [51]. For *R. amurensis*, as temperature increases, the pathogenic bacteria Enteroles and Enteroceae under Proteobacteria significantly increase, and the pathogenic bacteria *Pseudoramibacter Eubacterium* and Alphaproteo under Actinobacteria also significantly increase. Concurrently, an increase in temperature is accompanied by a decrease in the probiotic *Bifidobacterium* under Actinobacteria (Figure 4B). In the context of decreased total energy intake and increased disease risk, maintaining health becomes a priority for the host to ensure the smooth progression of subsequent critical life history events (such as reproduction and hibernation), which may lead to a sacrifice in its antioxidant capacity [111,119,120,121,122]. This unified response at the phylum level and specific response at the genus level reveal the hierarchical regulation characteristics of microbial communities. Given that numerous studies have confirmed that the regulation of gut microbiota communities on the host is related to the gut–brain axis, daily fluctuating temperature may regulate the physiological state of the host by altering the gut microbiota community dominated by Actinobacteria [48,123,124]. However, the differences in regulation between the two cold-adapted brown frog species may reflect the unique adaptability of different species to environmental changes, with *R. dybowskii* developing a more effective microbial community buffering system in response to contrasting peak daily habitat temperatures [39,60]. Additionally, in natural habitats, during these periods (emergence and preceding hibernation), the two brown frogs may still engage in a small amount of feeding activities, which may differ to some extent from the complete fasting state simulated in the experiment [62]. However, the consistency of all experimental treatments also reflects to some extent what might occur in their natural state.

Despite the systematic elucidation of the regulatory mechanisms of temperature on the physiological-microbial system of brown frogs in this study, the impact of ambient temperature on amphibians exhibits multidimensional and cross-scale characteristics, involving complex interactions among physiology, immunity, and microbial communities. Consequently, several pressing scientific issues remain to be addressed: (1) The challenges posed by extreme climatic events such as diurnal temperature variation, alterations in day–night length, and spring climate traps to the survival of amphibians in the context of global warming [40,125,126,127]; (2) The potential influence of micro-environmental temperature gradients within hibernation nests on microbial composition through epigenetic regulation, necessitating cross-scale observations [39]; (3) The unresolved synergistic defense mechanisms between amphibian skin antimicrobial peptides and gut microbiota, particularly the molecular diversity of the antimicrobial peptide and its complementary effects with lysozyme, which merit further investigation [128,129]. These studies can better predict and respond to potential changes in ecosystems, ensuring the stability of biodiversity. This not only holds significant importance for brown frog populations but also provides a scientific basis for the conservation of other brown species.

## 5. Conclusions

In response to contrasting peak daily habitat temperatures (4 °C vs. 20 °C) representing pre- and post-hibernation thermal conditions, two cold-adapted brown frog species in high-latitude cold regions have evolved differentiated physiological–microbial interaction strategies. *R. dybowskii* shows efficient immune system activation associated with the synergistic effect of a reduction in pathogenic bacteria in the gut microbiota and the inhibition of immunometabolic pathways. However, the decline in the abundance of probiotics exacerbates energy metabolism imbalance, ultimately leading to increased levels of oxidative stress. *R. amurensis* encounters more complex regulatory challenges: a significant proliferation of pathogenic bacteria within the gut microbiota, a decreased abundance of beneficial bacteria, and a loss of diversity. While these changes may enhance immune responses in the short-term, they further amplify oxidative damage due to the trade-off effect in energy allocation.

The aforementioned differences suggest that *R. dybowskii*, which has a lower cold tolerance, exhibits functional plasticity in its gut microbiota, which may contribute to maintaining immune function but at the cost of increased oxidative stress. In contrast, *R. amurensis*, which demonstrates a stronger cold tolerance, incurs higher metabolic costs due to the instability in its gut microbiota structure of thermal environments. Although both strategies exhibit interspecific differentiation, which may reflect divergent adaptive mechanisms and potentially contribute to niche partitioning, they may have potential negative impacts on population viability. This study provides a new perspective for analyzing the niche differentiation mechanisms of amphibians under climate change and suggests that future research should focus on cross-scale regulatory networks such as micro-environmental temperature gradients and host-microbiota co-defense.

## Data Availability

The raw reads were deposited in the SRA database within the BioProject (Accession Numbers: PRJNA1254415 and PRJNA1254418).

## Supplementary Materials

The following supporting information can be downloaded at: https://www.mdpi.com/article/10.3390/ani15243604/s1, Figure S1. Experimental animal facilities, environmental temperature conditions, and the degree of PHA response. (A) The experimental animal cages; (B) The peak daily habitat temperatures in the local area during the period after emergence and prior to hibernation (April and October) in 2019–2022; (C) Temporal variation in PHA response in *R. dybowskii*; (D) Temporal variation in PHA response in *R. amurensis*. Significant group differences are denoted by asterisks. * stands for 0.01 < *p* ≤ 0.05, ** stands for 0.001 < *p* ≤ 0.01. Figure S2. The seven alpha diversity indices of *R. dybowskii* (A) and *R. amurensis* (B) at different temperatures. Significant group differences are denoted by asterisks. ** stands for 0.001 < *p* ≤ 0.01. Figure S3. The beta diversity index and NMDS of *R. dybowskii* (A,B) and *R. amurensis* (C,D) at different temperatures. Figure S4. The bacteria composition of intestinal flora of *R. dybowskii* and *R. amurensis* at different levels (A,B): phylum; (C,D): class; (E,F): order; (G,H): family; (I,J): genus; (K,L): species. Significant group differences are denoted by asterisks. ** stands for 0.001 < *p* ≤ 0.01. Figure S5. The group of Venn of *R. dybowskii* (A) and *R. amurensis* (B). Table S1. Effects of sex on various indicators under contrasting peak daily habitat temperatures.

## Abbreviations

The following abbreviations are used in this manuscript:

ANOVA	Analysis of variance
ASVs	Amplicon sequence variants
BMI	Body mass index
CAT	Catalase
FDR	False discovery rate
GPCRs	G protein-coupled receptors
KEGG	Kyoto Encyclopedia of Genes and Genomes
LDA	Linear discriminant analysis
LEfSe	Linear discriminant analysis effect size
LSZ	Lysozyme
Lym	Lymphocytes
MDA	Malondialdehyde
NMDS	Nonmetric multidimensional scaling
PBS	Phosphate buffered saline
PHA	Phytohemagglutinin
PICRUSt2	Phylogenetic investigation of communities by reconstruction of unobserved states
POS	Preparation for oxidative stress
ROS	Reactive oxygen species
SCFAs	Short-chain fatty acids
SOD	Superoxide dismutase
Treg	T regulatory
