# Supplementary material for: The Immune-Antioxidant Trade-Off Mediated by Actinobacteria Drives Niche Differentiation: Physiological and Gut Microbiota Responses of Two Cold-Adapted Brown Frog Species to Contrasting Peak Daily Habitat Temperatures
Source: Animals (Basel). 2025 Dec 15;15(24):3604. doi: 10.3390/ani15243604 (PMC12729615; doi:10.3390/ani15243604)
Supplement: Supplementary file 1 [file animals-15-03604-s001.zip › animals-4024821-supplementary.pdf]

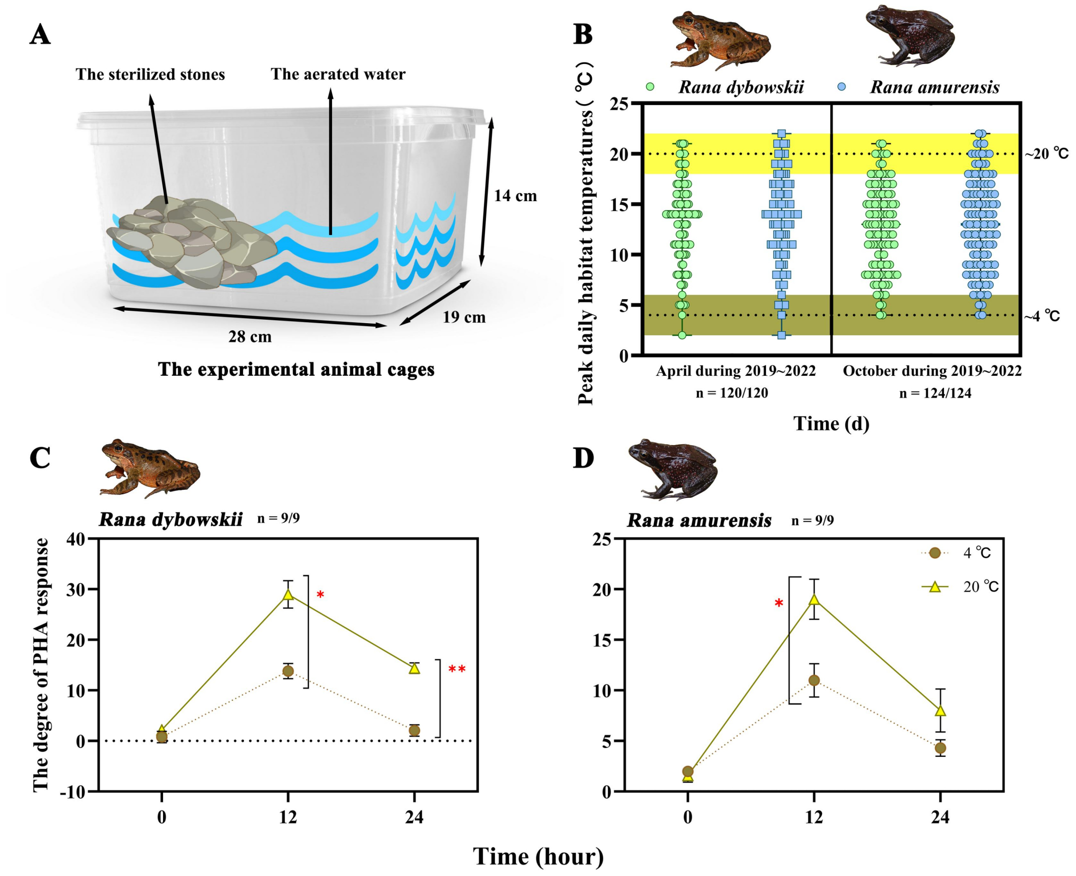

Figure S1. Experimental animal facilities, environmental temperature conditions, and the degree of PHA response. (A) The experimental animal cages; (B) The peak daily habitat temperatures in the local area during the period after emergence and prior to hibernation (April and October) in 2019-2022; (C) Temporal variation in PHA response in *R. dybowskii*; (D) Temporal variation in PHA response in *R. amurensis*. Significant group differences are denoted by asterisks. * stands for $0.01 < p \leqslant 0.05$, ** stands for $0.001 < p \leqslant 0.01$.

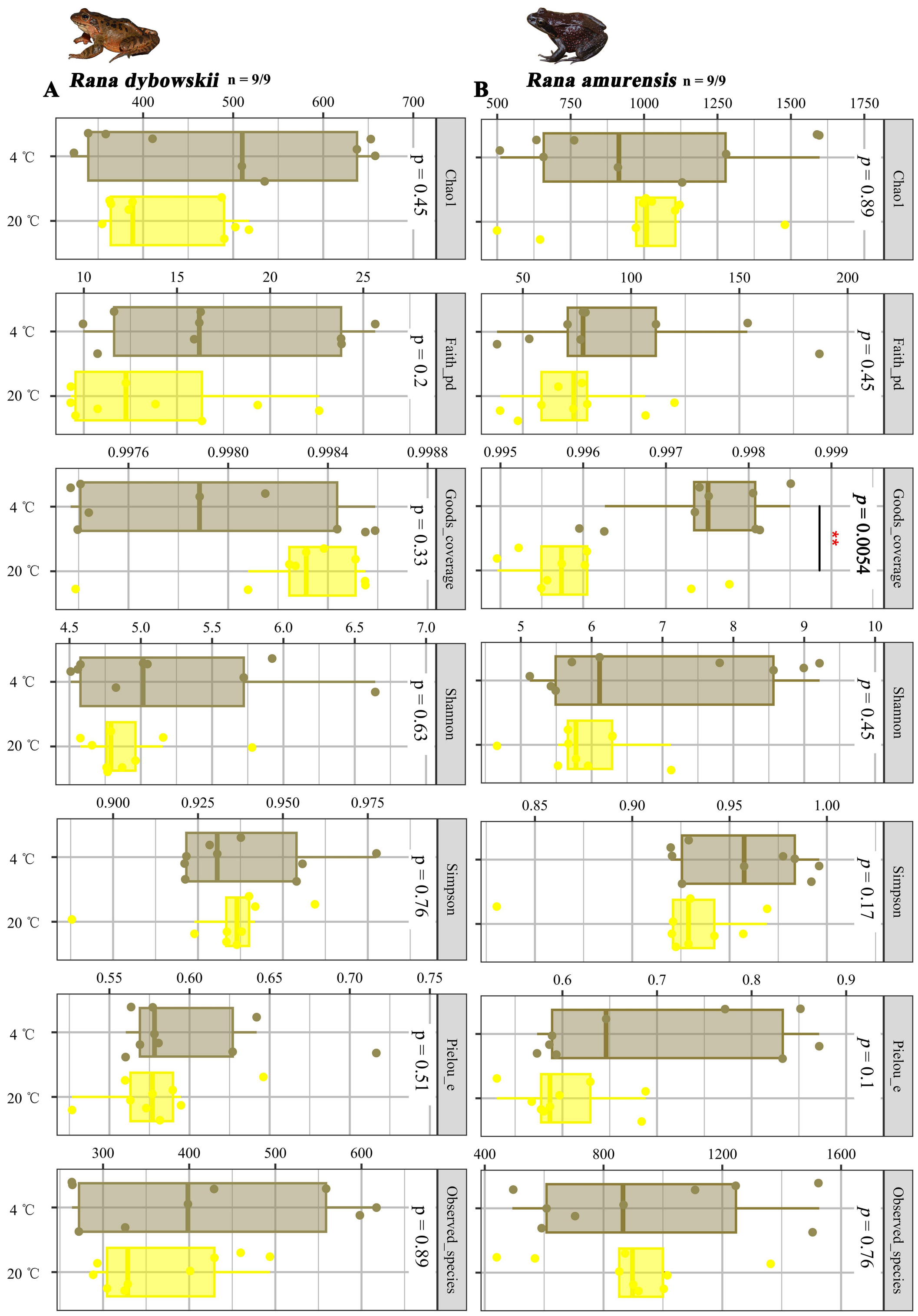

Figure S2. The seven alpha diversity indices of *R. dybowskii* (A) and *R. amurensis* (B) at different temperatures. Significant group differences are denoted by asterisks. ** stands for $0.001 < p \leqslant 0.01$.

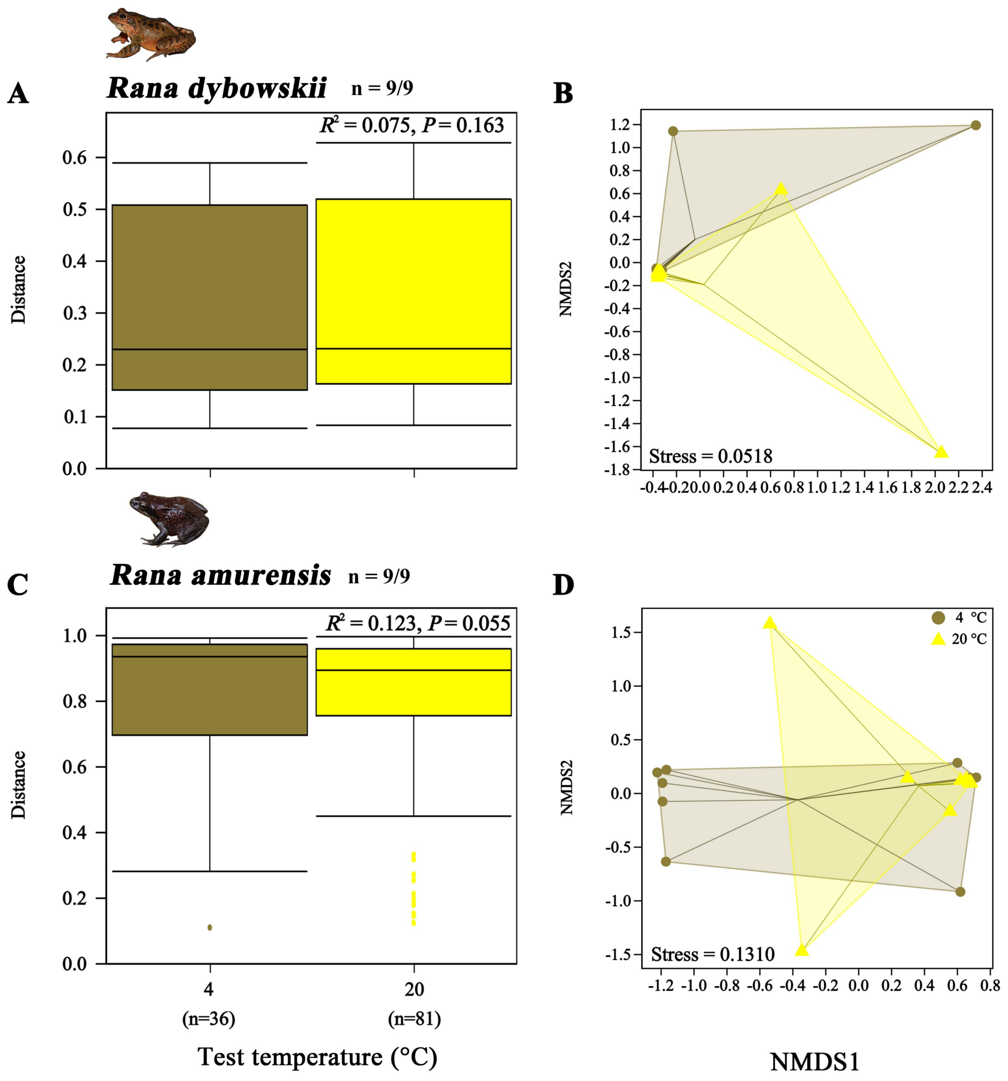

Figure S3. The beta diversity index and NMDS of *R. dybowskii* (A,B) and *R. amurensis* (C,D) at different temperatures.

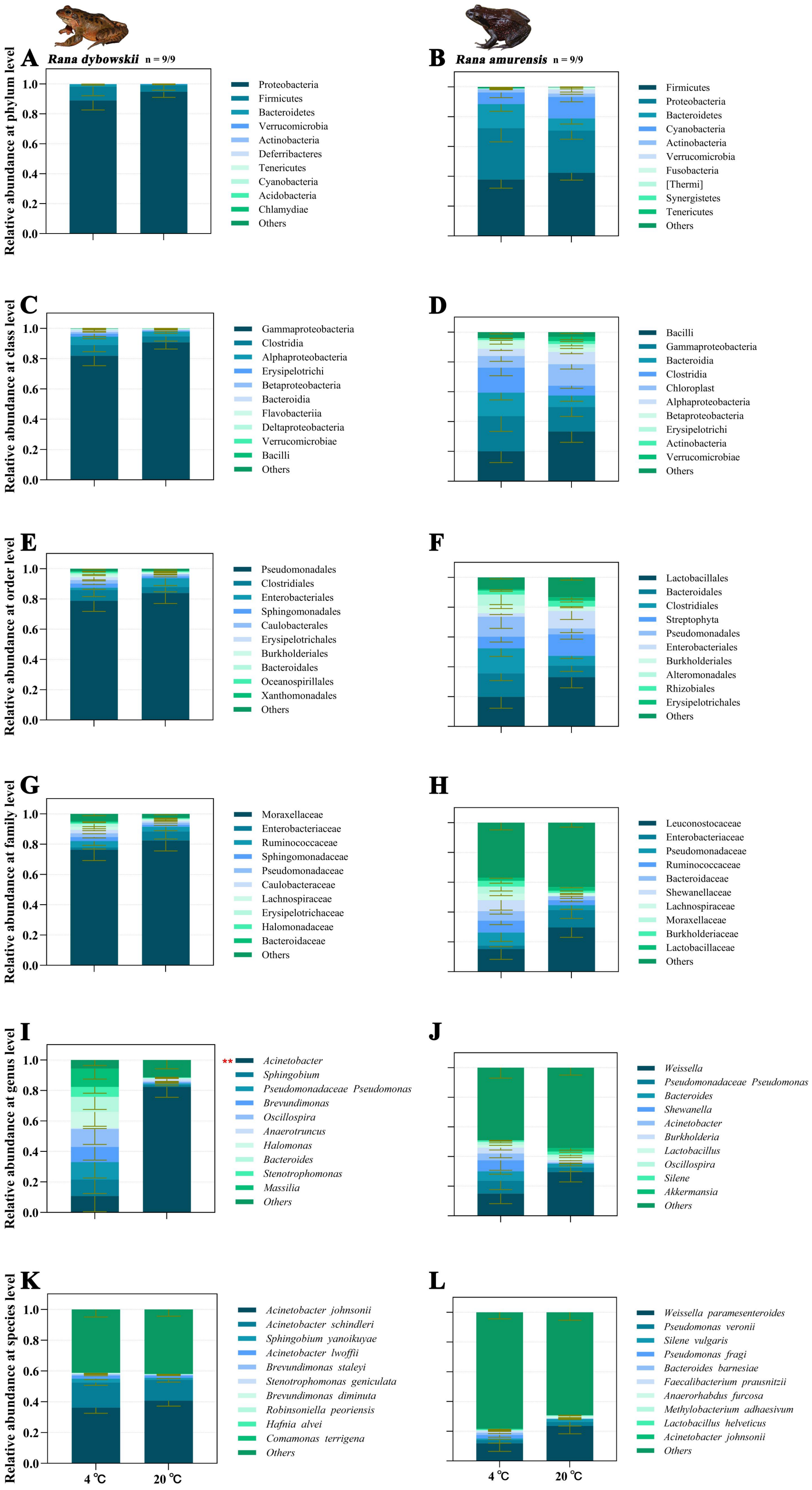

Figure S4. The bacteria composition of intestinal flora of *R. dybowskii* and *R. amurensis* at different levels (A,B): phylum; (C,D): class; (E,F): order; (G,H): family; (I,J): genus; (K,L): species. Significant group differences are denoted by asterisks. ** stands for $0.001 < p \leqslant 0.01$.

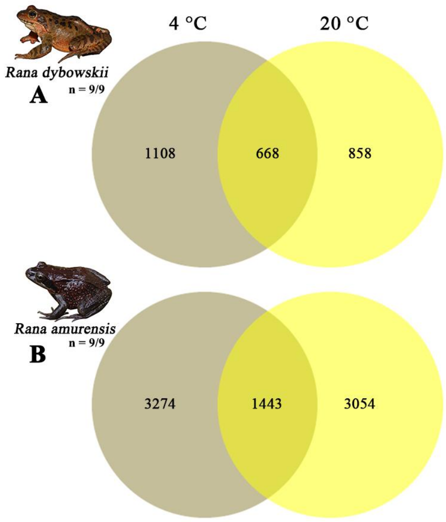

Figure S5. The group of Venn of *R. dybowskii* (A) and *R. amurensis* (B). Table S1. Effects of sex on various indicators under contrasting peak daily habitat temperatures.

Table S1. Effects of sex on various indicators under contrasting peak daily habitat temperatures.

Treat	Species	Indicators	*F* value	*P* value
Sex	*Rana dybowskii*	The degree of PHA response	0.507	0.550
		LSZ	1.456	0.247
		Lym	2.470	0.167
		SOD	0.010	0.922
		CAT	0.530	0.479
		MDA	0.005	0.944
		The goods coverage	-0.245	0.806
		Relative abundance at *Acinetobacter*	2.909	0.110
	Rana amurensis	The degree of PHA response	1.950	0.297
		LSZ	0.344	0.567
		Lym	8.172	0.029
		SOD	0.072	0.792
		CAT	1.634	0.222
		MDA	3.603	0.078
		The goods coverage	0.000	1.000
		Relative abundance at *Acinetobacter*	0.079	0.783
Sex * Temperature	*Rana dybowskii*	The degree of PHA response	5.231	0.149
		LSZ	0.138	0.715
		Lym	0.085	0.781
		SOD	0.011	0.920
		CAT	0.498	0.492
		MDA	0.741	0.404
		The goods coverage	0.000	1.000
		Relative abundance at *Acinetobacter*	3.614	0.078
	Rana amurensis	The degree of PHA response	0.190	0.705
		LSZ	0.360	0.558
		Lym	0.001	0.972
		SOD	1.363	0.204
		CAT	0.611	0.448
		MDA	0.476	0.501
		The goods coverage	-0.980	0.327
		Relative abundance at *Acinetobacter*	4.550	0.051
